# Dehydroepiandrosterone administration modulates endothelial and neutrophil adhesion molecule expression *in vitro*

**DOI:** 10.1186/cc4986

**Published:** 2006-07-19

**Authors:** Tanja Barkhausen, Britt-Mailin Westphal, Claudia Pütz, Christian Krettek, Martijn van Griensven

**Affiliations:** 1 Department of Trauma Surgery, Hanover Medical School, Carl-Neuberg Strasse, D-30625 Hannover, Germany; 2 Ludwig Boltzmann Institute for Experimental and Clinical Traumatology, Donaueschingenstrasse, A-1200 Vienna, Austria

## Abstract

**Introduction:**

The steroid hormone dehydroepiandrosterone (DHEA) exerts protecting effects in the treatment of traumatic and septic complications in several animal models. This effect goes along with reduced amounts of infiltrating immune cells in organs such as lung and liver. However, the underlying mechanisms of DHEA action are still not known. Adhesion molecules are important for the extravasation of neutrophils into organs where they may exhibit detrimental effects. Therefore, we investigated the *in vitro *effect of DHEA on the expression pattern of adhesion molecules of human endothelial cells and neutrophils.

**Methods:**

Endothelial cells derived from human umbilical cord were subjected to an lipopolysaccharide (LPS) challenge. DHEA was administered in two different concentrations, 10^-5 ^M and 10^-8 ^M, as a single stimulus or in combination with LPS challenge. After two, four and 24 hours, fluorescence activated cell sorter (FACS) analysis for vascular cell adhesion molecule-1, intercellular adhesion molecule-1 and E-selectin was performed. Neutrophils were freshly isolated from blood of 10 male healthy volunteers, stimulated the same way as endothelial cells and analyzed for surface expression of L-selectin, CD11b and CD18.

**Results:**

In the present study, we were able to demonstrate effects of DHEA on the expression of every adhesion molecule investigated. DHEA exhibits opposite effects to those seen upon LPS exposure. Furthermore, these effects are both time and concentration dependent as most DHEA specific effects could be detected in the physiological concentration of 10^-8 ^M.

**Conclusion:**

Thus, we conclude that one mechanism by which DHEA may exert its protection in animal models is via the differential regulation of adhesion molecule expression.

## Introduction

Trauma and sepsis are the leading causes of death in developed countries; incidence of mortality in septic patients could reach as high as 30% [1,2]. The reasons for this high incidence are very complex. After multiple trauma and/or sepsis the immune system becomes highly activated. Once the inflammatory cascade is initiated, it often results in a systemic inflammatory reaction that involves a variety of body systems, for example, the complement system [3,4], the coagulation system [5,6] and the bradykinin-kinin system [7,8]. After a few days, this physiological process will be resolved in some patients and they will convalesce, while in others it could lead to death. Despite years of research, the exact mechanisms underlying these different responses are still unknown.

The extravasation of leukocytes from the vascular bed into surrounding tissues and organs is part of the host defense mechanisms against invading pathogens. However, it could be one of the key factors contributing to organ failure and death in cases of disturbed body homeostasis. Activated by cytokines and chemokines, leukocytes and endothelial cells express distinct adhesion molecules on their cell surfaces [9]. These adhesion molecules enable the deceleration of blood cells on the endothelial layer in order to enable subsequent diapedesis. In the first phase of the extravasation process, selectins such as L-selectin on leukocytes and E- and P-selectin on endothelial cells lead to a loose connection that permits tethering and rolling of leukocytes on the endothelium under hydrodynamic shear [10]. Stable attachments between leukocytes and endothelial cells take place through interactions of integrins like CD18/CD11b, expressed on leukocytes [11], with their immunoglobulin-like ligands, such as intercellular adhesion molecule (ICAM)-1 and vascular cell adhesion molecule (VCAM)-1, which are expressed on endothelium [11,12]. The importance of adhesion molecules in traumatic and septic diseases has been widely recognized. A recent study by our group performed in ICAM-1 knockout mice demonstrated a significant reduction in mortality after trauma and sepsis [13,14]. Similar beneficial results can be obtained from studies inhibiting other adhesion molecules, such as L-selectin [15,16], P-selectin [17,18], E-selectin [19], CD11b [20,21] and CD18 [22]. Maekawa and colleagues [23] demonstrated that increases in expression levels of L-selectin, sL-selectin and CD11b correlate with injury severity after trauma. Thus, adhesion molecules seem to have an influence on the outcome and severity of complications after trauma and sepsis and are, therefore, interesting candidates for medication and drug targets.

Previous studies by our group dealing with the therapeutic effect of the steroid hormone dehydroepiandrosterone (DHEA) revealed that it is protective in a murine model of combined trauma and sepsis [24-27]. DHEA is the most abundant steroid hormone of the body and is a precursor of sexual hormones, such as 7-β-estradiol and 5-α-dihydrotestosterone. Additionally, we demonstrated that DHEA administration resulted in a reduced amount of granulocyte infiltration into organs [28].

Because of our previous findings concerning neutrophil extravasation in DHEA treated mice, we postulate that DHEA has either a direct or an indirect effect on the expression of adhesion molecules on leukocytes or endothelial cells. To investigate this phenomenon, we performed cell culture experiments with granulocytes and endothelial cells and determined expression levels of adhesion molecules during DHEA treatment after endotoxin (lipopolysaccharide (LPS)) challenge. LPS was chosen to mimic a 'septic' state in the cell environment. Experiments using DHEA treatment after LPS challenge should reveal if DHEA is able to attenuate inflammatory LPS effects.

## Materials and methods

### Endothelial cell culture and stimulation

The study was approved by the ethical committee of Hannover Medical School.

Endothelial cells were isolated and cultured from human umbilical cord vein, and are designated as human umbilical vein endothelial cells (HUVECs; *n *= 7). Fresh umbilical cords of 10 to 30 cm were prepared by inserting cannulas in both ends of the umbilical cord vein. Via the cannula, the vein was rinsed with sterile, prewarmed cord buffer (3.0 mM NaH_2_PO_4_·H_2_O; 9.0 mM Na_2_HPO_4_·2H_2_O; 140 mM NaCl; 8.5 mM MgCl_2_·6H_2_O; 11 mM glucose), filled with collagenase (0.04% w/v in cord buffer; Gibco, Grand Island, USA) solution and sealed at both ends. Enzyme incubation was conducted for 30 minutes at 37°C to release endothelial cells from the extracellular matrix. After incubation, collagenase solution containing endothelial cells was eluted from the umbilical cord, cells were washed by centrifugation in PBS and plated in T25 cell culture bottles (Cell+, Greiner, Frickenhausen, Germany) in Endothelial Cell Culture Medium (Promocell, Heidelberg, Germany). Cells were grown to sub-confluence and passaged by enzymatic detachment with Trypsin/EDTA (Biochrom, Berlin, Germany). After another washing step, 2.5 × 10^5 ^cells were seeded and expanded in passage 1 into T75 cell culture bottles. Cells from passages 1 to 3 were used for the experiments.

For the experimental procedure, 5 × 10^4 ^cells were seeded into 6-well plates and grown to sub-confluence. In the sub-confluential state, cells were exposed to 100 ng/ml LPS obtained from *Escherichia coli *O111:B7 (Sigma, Deisenhofen, Germany). Additionally, cells were treated upon LPS incubation with two different DHEA concentrations (10^-5 ^M or 10^-8 ^M; Sigma, Deisenhofen, Germany); these concentrations were also used as single stimuli to determine DHEA specific effects. Unstimulated cells were used as internal controls (Table 1). Experiments were performed for two, four, and 24 hours.

### Polymorph nuclear neutrophil isolation and stimulation

EDTA-blood was drawn from healthy male volunteers (*n *= 10) with an average age of 28 ± 5 years. Volunteers with any kind of disease, especially persons suffering from systemic inflammatory disorders, were excluded from the study. Blood was diluted 1:2 and a first separation step was performed by Ficoll gradient centrifugation. Neutrophils in the pellet of the gradient were separated from erythrocytes by further steps consisting of dextran sedimentation and hypotonic lysis. Cells (10^6^/ml) were seeded into 24-well plates (Greiner) using RPMI 1640 medium (Biochrom) containing 10% serum of the respective volunteer. Stimulation was performed immediately after isolation as described for endothelial cells with stimulation times of two, four and 24 hours. Again, untreated cells were used as internal controls.

### Flow cytometry

For flow cytometry analysis, antibodies against VCAM-1, ICAM-1, E-selectin, L-selectin, CD11b and CD18 were used. Human endothelial cells were investigated for the expression of VCAM-1, ICAM-1 and E-selectin. Human polymorph nuclear neutrophils were stained with anti-L-selectin, anti-CD11b and anti-CD18. All antibodies used for flow cytometric analysis were obtained from Becton Dickinson (San Jose, CA, USA), except the CD62L specific antibody, which was purchased from BenderMedSystems (Vienna, Austria). Stimulation was stopped by adding 1 ml of ice-cold PBS to the cell suspension. Endothelial cells were detached using a cell scraper (Greiner). Cells were transferred into round bottom polypropylene tubes (Becton Dickinson). After pelleting by centrifugation, cells were washed again and resuspended in 100 μl PBS containing 10 μl of the respective antibody solution. Cells were incubated for 30 minutes at 4°C. Subsequently, cells were washed with PBS and resuspended in 300 μl PBS for flow cytometric analysis. Analysis was conducted on a FACSCalibur (Becton Dickinson) with individual settings for each antibody utilizing Cell Quest Pro Software (Becton Dickinson). Unstained cells were used to discriminate autofluorescence and to adjust forward and side scatter. Amounts of positive cells were given in percent. For further analysis, relative expression was calculated by the ratio of stimulated cells to unstimulated cells and presented in percent (100% = expression level of unstimulated cells).

### Statistics

Statistical analysis was performed using a standard software application (SPSS, SPSS Inc., Chicago, IL, USA). Comparisons between groups were performed using one-way analysis of variances (ANOVA) followed by Student *t *test. Probability values less then 0.05 were considered statistically significant. The data are expressed as mean ± standard error of the mean.

## Results

### Adhesion molecule expression of HUVECs *in vitro*

#### VCAM-1 expression

**Figure 1:**
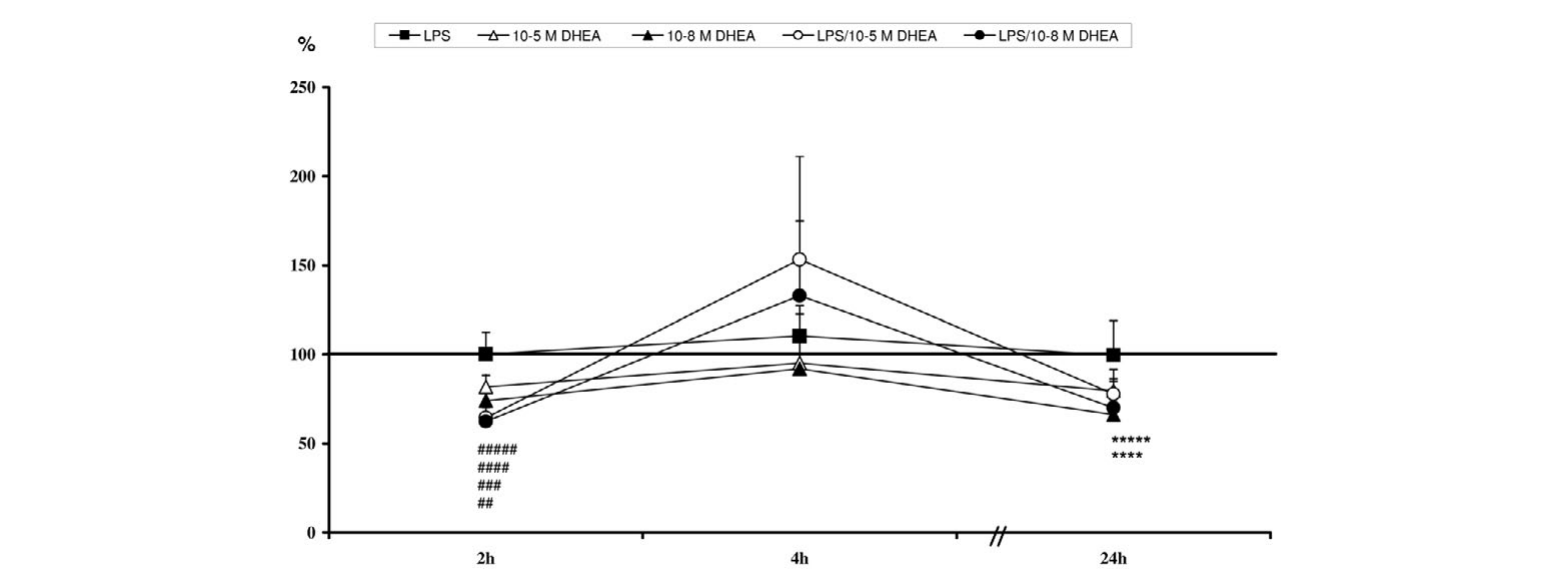
Relative vascular cell adhesion molecule-1 expression levels: ^##^10^-5 ^M dehydroepiandrosterone (DHEA) significant compared to unstimulated and lipopolysaccharide (LPS); ^###^10^-8 ^M DHEA significant compared to unstimulated and LPS; ^####^LPS/10^-5 ^M DHEA significant compared to unstimulated and LPS; ^#####^LPS/10^-8 ^M DHEA significant compared to unstimulated and LPS; ****LPS/10^-5 ^M DHEA significant compared to unstimulated; *****LPS/10^-8 ^M DHEA significant compared to unstimulated.

LPS had no modulating effects on VCAM-1 expression in this experimental setting (Table 2, Figure 1).

In contrast, DHEA of both concentrations, in single stimulation experiments as well as in experiments using DHEA treatment after LPS challenge, induced a down-regulation of membrane-bound VCAM-1 after two hours (Table 2, Figure 1) compared to controls and LPS treated samples. Expression levels tended to normalize in single DHEA treated samples until the final observation time (Table 2, Figure 1). This down-regulation was also detectable after 24 hour treatment with DHEA after LPS challenge (Table 2, Figure 1).

Interestingly, all groups showed expression peaks in the time course after four hours, with no significant differences between the groups (Table 2, Figure 1).

#### ICAM-1 expression

**Figure 2:**
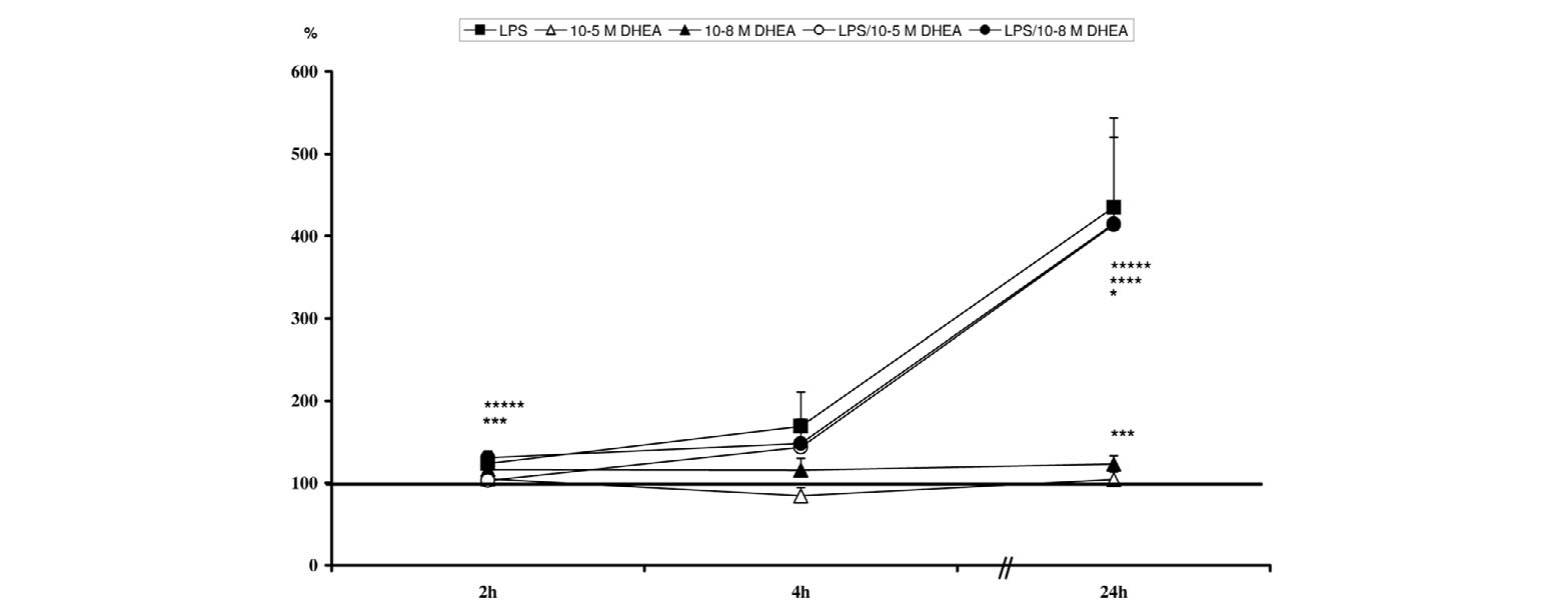
Relative intercellular adhesion molecule-1 expression levels: *lipopolysaccharide (LPS) significant compared to unstimulated; ***10^-8 ^M dehydroepiandrosterone (DHEA) significant compared to unstimulated; ****LPS/10^-5 ^M DHEA significant compared to unstimulated; *****LPS/10^-8 ^M DHEA significant compared to unstimulated.

LPS induced a steady increase of ICAM-1 expression over the time course in all groups, with peak ICAM-1 expression levels after 24 hours. Significant differences compared to controls were measurable two hours after treatment with LPS/10^-8 ^M DHEA, and for all LPS stimulated groups after 24 hours (Table 3, Figure 2). A significant up-regulation of ICAM-1 occurred with 10^-8 ^M DHEA alone after two and 24 hours but, in contrast to LPS, alterations caused by 10^-8 ^M DHEA were not so pronounced. ICAM-1 expression was not affected by 10^-5 ^M DHEA, either alone or after LPS challenge (Table 3, Figure 2).

#### E-selectin expression

**Figure 3:**
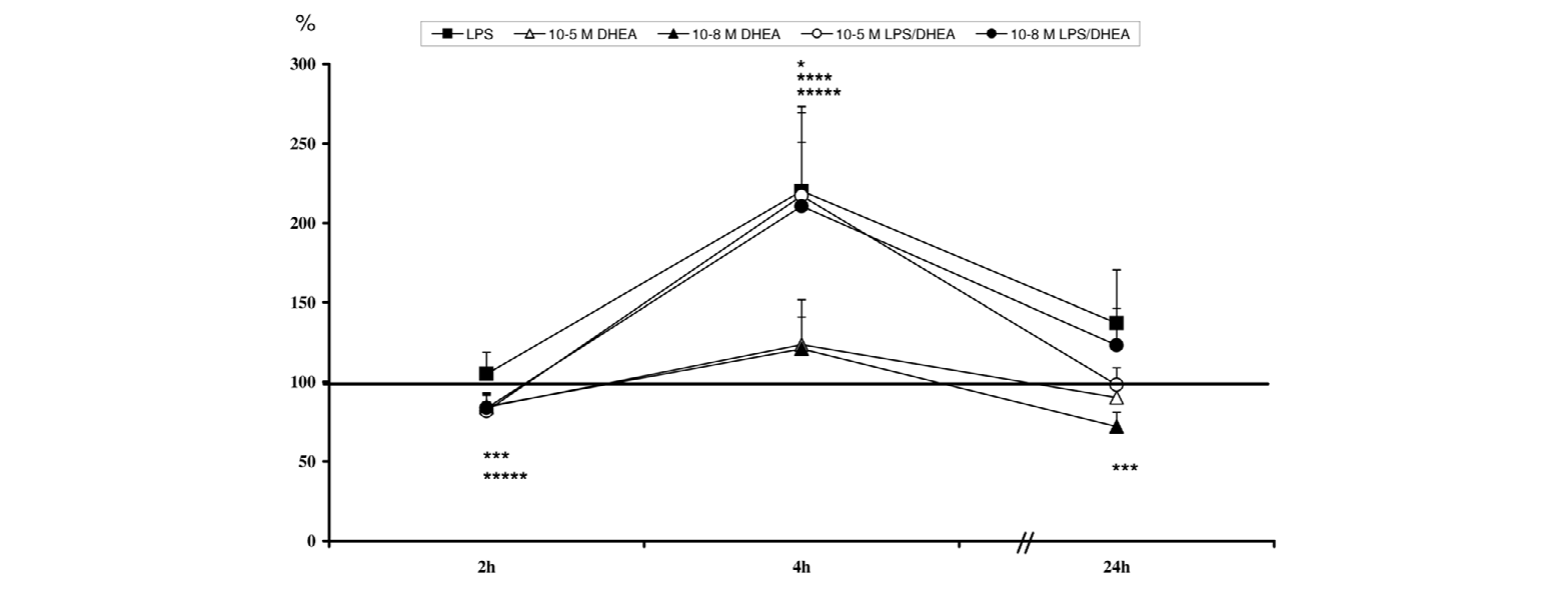
Relative E-selectin expression levels: *lipopolysaccharide (LPS) significant compared to unstimulated; ***10^-8 ^M dehydroepiandrosterone (DHEA) significant compared to unstimulated; ****LPS/10^-5 ^M DHEA significant compared to unstimulated; *****LPS/10^-8 ^M DHEA significant compared to unstimulated.

After two hours of stimulation, incubation with 10^-8 ^M DHEA resulted in a reduction of E-selectin expression. This effect was detected after single stimulation as well as after stimulation with LPS and DHEA (Table 4, Figure 3).

E-selectin expression levels peaked at four hours in all groups, with significantly up-regulated values compared to unstimulated controls in all LPS treated groups (Table 4, Figure 3). These levels seemed to normalize after 24 hours. Single treatment with 10^-8 ^M DHEA caused a significantly reduced expression of E-selectin (Table 4, Figure 3).

### Adhesion molecule expression of human neutrophils *in vitro*

#### L-selectin expression

**Figure 4:**
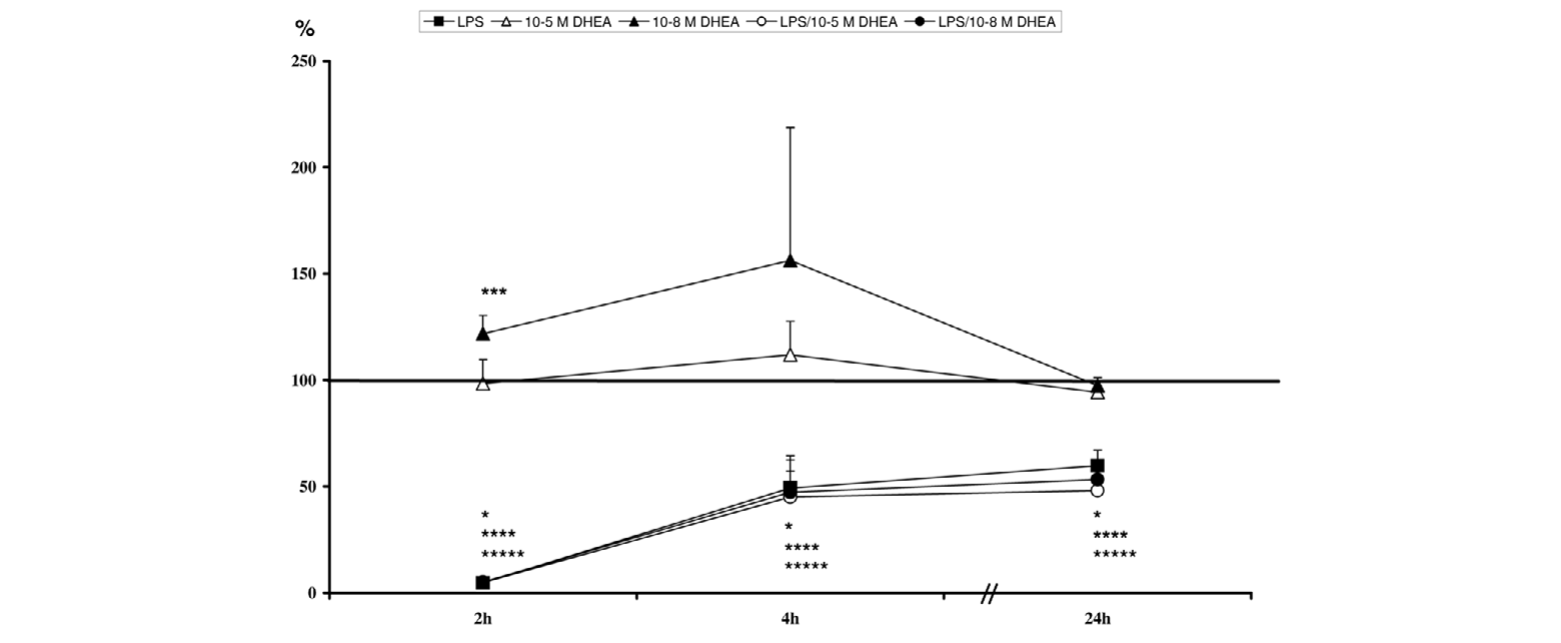
Relative L-selectin expression levels: *lipopolysaccharide (LPS) significant compared to unstimulated; ***10^-8 ^M dehydroepiandrosterone (DHEA) significant compared to unstimulated; ****LPS/10^-5 ^M DHEA significant compared to unstimulated; *****LPS/10^-8 ^M DHEA significant compared to unstimulated.

During exposure to LPS, L-selectin is rapidly shed from cell surfaces. After two hours, its expression levels in LPS treated groups were significantly reduced, with levels tending to zero (Table 5, Figure 4). Levels started to recover in the LPS treated groups after four and 24 hours, but were still significantly decreased (Table 5, Figure 4).

In contrast, stimulation with 10^-8 ^M DHEA caused an up-regulation of L-selectin expression after two hours (*p* < 0.05). Levels showed no significant changes with 10^-5 ^M DHEA and with 10^-8 ^M DHEA at the later measurement points (Table 5, Figure 4).

#### CD11b expression

**Figure 5:**
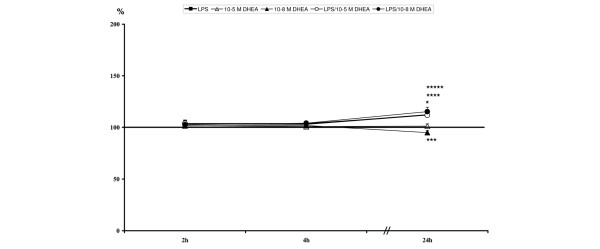
Relative CD11b expression levels: *lipopolysaccharide (LPS) significant compared to unstimulated; ***10^-8 ^M dehydroepiandrosterone (DHEA) significant compared to unstimulated; ****LPS/10^-5 ^M DHEA significant compared to unstimulated; *****LPS/10^-8 ^M DHEA significant compared to unstimulated.

At two and four hours after stimulation, CD11b expression was not affected by either LPS or DHEA at any concentration (Table 6, Figure 5).

Expression of CD11b was significantly up-regulated in all samples stimulated with LPS (with and without DHEA treatment) compared to unstimulated controls at 24 hours (Table 6, Figure 5). In contrast, single treatment with 10^-8 ^M DHEA resulted in a significant decrease in CD11b expression (Table 6, Figure 5).

#### CD18 expression

**Figure 6:**
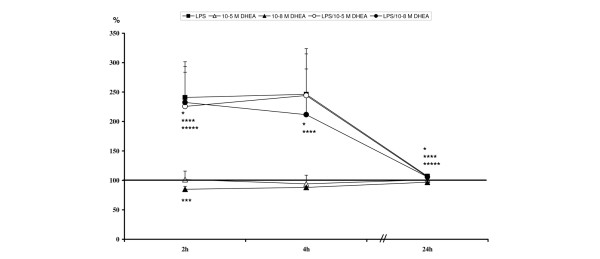
Relative CD18 expression levels: *lipopolysaccharide (LPS) significant compared to unstimulated; ***10^-8 ^M dehydroepiandrosterone (DHEA) significant compared to unstimulated; ****LPS/10^-5 ^M DHEA significant compared to unstimulated; *****LPS/10^-8 ^M DHEA significant compared to unstimulated.

At two hours, all LPS stimulated groups showed significant increases in CD18 expression levels (Table 7, Figure 6). This effect was also observed after four hours. After 24 hours, CD18 expression tended to recover in the LPS treated groups but was still significantly increased compared to unstimulated controls (Table 7, Figure 6).

In contrast to LPS, a single treatment with 10^-8 ^M DHEA resulted in an early decrease of CD18 expression after two hours, but after 24 hours DHEA had not affected CD18 expression compared to unstimulated controls (Table 7, Figure 6).

## Discussion

The steroid hormone DHEA has been shown to be beneficial in animal experiments of trauma or sepsis [24-27]. Previous studies by our group revealed that these beneficial effects are concomitant with a reduced amount of infiltrating neutrophils in distinct tissue sites, for example, lung tissue [28]. As neutrophil extravasation is mainly caused by adhesion molecules, we suggested that DHEA has a specific effect on adhesion molecule expression. In the present study we demonstrate that DHEA has distinct *in vitro *effects on surface expression patterns of adhesion molecules of endothelial and neutrophil origin. Interestingly, we observed that the mode of DHEA action is different with different adhesion molecules. Additionally, we detected time-dependent effects as well as DHEA concentration-dependent effects.

In the present study, we used two different concentrations of DHEA (10^-5 ^M and 10^-8 ^M). As a first important finding, the strongest effects of DHEA occurred with a DHEA concentration of 10^-8 ^M compared to a concentration of 10^-5 ^M. A direct effect of 10^-5 ^M DHEA could only be detected on VCAM-1 expression. All other DHEA-dependent changes were observed after treatment with 10^-8 ^M DHEA. This result is interesting because a concentration of 10^-8 ^M resembles the physiological DHEA concentration. Normal DHEA serum levels show values of 10 nmol/l [29]. Thus, at least in the current experimental setting, super-physiological concentrations of DHEA are less efficacious than the physiological range.

Adhesion molecules are influenced under septic conditions. LPS, a component found in the outer membrane of Gram-negative bacteria, is one of the key players mediating septic effects. With LPS as the single inflammatory stimuli *in vitro*, adhesion molecules exhibit divergent time courses of expression. In this experimental setting investigating human neutrophils and endothelial cells, the time courses of expression were likewise dependent on the adhesion molecule studied. We observed constant increases in the expression patterns of ICAM-1 and CD11b, a peak expression after four hours of stimulation with LPS for E-selectin, and an early induction of CD18 followed by a down-regulation over the time course. In contrast, L-selectin was rapidly shed from cell surfaces but its levels recovered, although not to baseline levels, over the time course. Similar to our results, analogous adhesion molecule dependent time courses of expression can be observed after stimulation with other inflammatory molecules, such as tumour necrosis factor-α and interleukin-1β [30,31]. The physiological background for these distinct reaction types, such as shedding or up-regulation, is not yet clear and requires further investigation.

One interesting finding was that LPS exhibited no effect on VCAM-1 expression. This was unexpected as several results from the literature refer to increases in VCAM-1 expression after LPS treatment [32-34]. Explanations for this discrepancy may be different culture conditions and different LPS concentrations; in the studies mentioned above, different medium types and serum concentrations were used compared to our experimental setting. Additionally, the LPS concentration used in these studies were higher, ranging from 1 μg/ml to 20 μg/ml [32-34], compared to the present study, which used 100 ng/ml. However, the main focus of this study was the potential modulation of adhesion molecules by DHEA. Thus, we demonstrated that treatment of endothelial cells with DHEA in each setting results in an abrogation of VCAM-1 expression from the cell surface at the earliest measurement point (after two hours). Additionally, after 24 hours a reduction was still detectable, but only in the DHEA treated samples. Therefore, we suggest that a DHEA-dependent reduction of VCAM-1 with an enhancing function of LPS occurred. A similar decline in expression after DHEA treatment was observed for E-selectin at two and 24 hours. Additionally, DHEA administration resulted in a decrease of ICAM-1, CD11b and CD18 at varying time points. In contrast to these reductions induced by DHEA, we found a DHEA-triggered increase of L-selectin expression. This is of interest, as DHEA seems to reverse the LPS specific response of each adhesion molecule in all cases when comparing single stimulations. This means, in cases where LPS alone induces up-regulation, incubation with solely DHEA results in a decrease and vice versa. Thus, we conclude that there is a direct correlation between the modulating effect of DHEA and the inflammatory context.

The mode of action by which DHEA influences certain adhesion molecules may occur at the signal transduction level. DHEA is known to influence distinct signal transduction pathways, such as the phosphoinositide 3-kinase (PI3K)/Akt, p38 mitogen-activated protein kinase (MAPK) and glycogen synthase kinase (GSK)-3β pathways [35-37]. It is possible that DHEA acts as an inhibitor of these kinases through dephosphorylation via a DHEA-enhanced dual specificity protein phosphatase [37]. As adhesion molecules are themselves influenced by a variety of signal kinases, such as protein kinase C-δ, phosphoinositide 3-kinase, Src, p38, JNK, extracellular signal-regulated kinase (ERK)1/2, and glycogen synthase kinase-3 [38-44], an interrelationship between DHEA and adhesion molecule expression on the signal transduction level can be considered and is under further investigation.

Experiments using DHEA treatment after LPS challenge in this study were designed to determine whether DHEA is able to directly modulate or rather reverse LPS specific effects. The results show that DHEA does not have the ability to modulate LPS induced changes in adhesion molecule expression in this experimental setting. However, it must be taken into account that the study was performed in an *in vitro *environment. Thus, the results might be dependent on concentrations of LPS and DHEA. Additionally, important co-factors might be missing that are ordinarily available in an *in vivo *environment. Despite this, DHEA does modulate adhesion molecules when LPS is not present or has no influence itself, and an effect under physiological conditions can thus be speculated to occur.

## Conclusion

We have demonstrated that DHEA exhibits modulating effects on adhesion molecule expression of human endothelial cells and neutrophils in an *in vitro *environment. Furthermore, we found that modulating effects triggered by DHEA treatment were always opposite to the effects induced by LPS. However, these effects could not be detected when DHEA was applied after LPS challenge. Thus, DHEA was not able to reverse inflammatory effects *in vitro*. Nevertheless, we do conclude that one mechanism of action by which DHEA exerts protective effects is via the modulation of adhesion molecules as DHEA alone did affect adhesion molecule expression. In this experimental setting, cofactors that are essential for the modulation of inflammatory responses *in vivo *might have been missing.

## Key messages

• 	The steroid hormone DHEA has modulating properties on adhesion molecule expression in endothelial cells and neutrophils.

• 	DHEA is not able to reverse LPS specific effects on adhesion molecule expression *in vitro*.

• 	DHEA acts in a concentration-dependent manner, with most effects detectable with a physiological concentration of 10^-8 ^M.

## Abbreviations

DHEA = dehydroepiandrosterone; HUVEC = human umbilical vein endothelial cell; ICAM = intercellular adhesion molecule; LPS = lipopolysaccharide; PBS = phosphate buffered saline; VCAM = vascular cell adhesion molecule.

## Competing interests

The authors declare that they have no competing interests.

## Authors' contributions

TB was responsible for conception and design of the study, acquired data, did statistical analysis and interpreted results. BMW and CP carried out isolation and measurement of neutrophils. CK read the manuscript and supported the lab team. MvG conceived the study, and critically revised and helped to draft the manuscript. All authors read and approved the final manuscript.

## Figures and Tables

**Table 1:** Overview of the experimental setting: measurement times, treatment procedures and concentrations

Time	Treatment					
2 h	Control	LPS 100 ng/ml	DHEA 10^-5 ^M	DHEA 10^-8 ^M	LPS (100 ng/ml) + 10^-5 ^M DHEA	LPS (100 ng/ml) + 10^-8 ^M DHEA
4 h	Control	LPS 100 ng/ml	DHEA 10^-5 ^M	DHEA 10^-8 ^M	LPS (100 ng/ml) + 10^-5 ^M DHEA	LPS (100 ng/ml) + 10^-8 ^M DHEA
24 h	Control	LPS 100 ng/ml	DHEA 10^-5 ^M	DHEA 10^-8 ^M	LPS (100 ng/ml) + 10^-5 ^M DHEA	LPS (100 ng/ml) + 10^-8 ^M DHEA

**Table 2:** Values of relative vascular cell adhesion molecule-1 expression

Time	LPS	10^-5 ^M DHEA	10^-8 ^M DHEA	LPS/10^-5 ^M DHEA	LPS/10^-8 ^M DHEA
2 h	100.13 ± 12.31	**81.73 ± 6.44**	**74.02 ± 14.55**	**64.43 ± 8.14**	**62.35 ± 7.81**
4 h	110.32 ± 17.1	95.12 ± 27.51	91.93 ± 26.85	153.16 ± 57.9	132.82 ± 42.03
24 h	99.58 ± 19.32	79.56 ± 11.98	66.16 ± 18.23	**77.73 ± 8.58**	**70.05 ± 14.88**

Levels are calculated against unstimulated controls (= 100%) and are given in percent ± standard error of the mean. Highlighted values at 2 h are significant compared to unstimulated and lipopolysaccharide (LPS); highlighted values at 24 h are significant compared to unstimulated. DHEA, dehydroepiandrosterone.

**Table 3:** Values of relative intercellular adhesion molecule-1 expression

Time	LPS	10^-5 ^M DHEA	10^-8 ^M DHEA	LPS/10^-5 ^M DHEA	LPS/10^-8 ^M DHEA
2 h	123.53 ± 10.56	104.97 ± 3.84	**116.11 ± 4.33**	102.87 ± 5.88	**130.76 ± 8.41**
4 h	169.02 ± 41.34	84.33 ± 9.84	115.58 ± 14.38	143.49 ± 33.42	147.88 ± 29.86
24 h	**434.9 ± 108.6**	104.08 ± 9.08	**123.02 ± 10.2**	**413.36 ± 106.17**	**414.96 ± 105.43**

Levels are calculated against unstimulated controls (= 100%) and are given in percent ± standard error of the mean. Highlighted values are significant compared to unstimulated. DHEA, dehydroepiandrosterone; LPS = lipopolysaccharide.

**Table 4:** Values of relative E-selectin expression

	LPS	10^-5 ^M DHEA	10^-8 ^M DHEA	LPS/10^-5 ^M DHEA	LPS/10^-8 ^M DHEA
2 h	105.03 ± 13.63	84.03 ± 8.46	**84.64 ± 8.5**	81.75 ± 9.79	**83.62 ± 3.8**
4 h	220.11 ± 53.07	123.47 ± 17.39	120.87 ± 30.71	217.23 ± 52.27	210.59 ± 40.36
24 h	137.05 ± 33.49	90.15 ± 6.42	**71.9 ± 9.02**	98.13 ± 10.87	123.07 ± 23.38

Levels are calculated against unstimulated controls (= 100%) and are given in percent ± standard error of the mean. Highlighted values are significant compared to unstimulated. DHEA, dehydroepiandrosterone; LPS = lipopolysaccharide.

**Table 5:** Values of relative L-selectin expression

	LPS	10^-5 ^M DHEA	10^-8 ^M DHEA	LPS/10^-5 ^M DHEA	LPS/10^-8 ^M DHEA
2 h	**4.66 ± 1.47**	98.36 ± 11.3	121.83 ± 8.6	**4.81 ± 1.21**	**5.01 ± 1.63**
4 h	**49.25 ± 13.22**	111.88 ± 15.78	156.24 ± 62.53	**45.02 ± 12.12**	**47.25 ± 17.27**
24 h	**59.77 ± 7.33**	94.28 ± 2.87	97.4 ± 3.86	**48.08 ± 6.02**	**53.26 ± 7.38**

Levels are calculated against unstimulated controls (= 100%) and are given in percent ± standard error of the mean. Highlighted values are significant compared to unstimulated. DHEA, dehydroepiandrosterone; LPS = lipopolysaccharide.

**Table 6:** Values of relative CD11b expression

	LPS	10^-5 ^M DHEA	10^-8 ^M DHEA	LPS/10^-5 ^M DHEA	LPS/10^-8 ^M DHEA
2 h	103.53 ± 3.46	101.7 ± 2.61	101.45 ± 2.51	103.84 ± 3.38	102.61 ± 3.56
4 h	103.16 ± 2.37	100.85 ± 1.15	101.85 ± 1.58	103.96 ± 2.03	104.12 ± 1.9
24 h	**112.18 ± 3.48**	101.28 ± 2.19	**95.05 ± 1.85**	**111.82 ± 3.69**	**115.35 ± 4.19**

Levels are calculated against unstimulated controls (= 100%) and are given in percent ± standard error of the mean. Highlighted values are significant compared to unstimulated. DHEA, dehydroepiandrosterone; LPS = lipopolysaccharide.

**Table 7:** Values of relative CD18 expression

	LPS	10^-5 ^M DHEA	10^-8 ^M DHEA	LPS/10^-5 ^M DHEA	LPS/10^-8 ^M DHEA
2 h	**240.86 ± 62.38**	101.48 ± 14.28	**84.88 ± 5.02**	**225.73 ± 58.03**	**232.53 ± 60.76**
4 h	**246.21 ± 73.37**	94.1 ± 14.71	88.1 ± 12.69	**244.21 ± 70.64**	211.68 ± 77.56
24 h	**107.15 ± 2.98**	100.97 ± 1.31	96.83 ± 2.26	**105.91 ± 2.45**	**105.95 ± 2.46**

Levels are calculated against unstimulated controls (= 100%) and are given in percent ± standard error of the mean. Highlighted values are significant compared to unstimulated. DHEA, dehydroepiandrosterone; LPS = lipopolysaccharide.
